# IKKε-deficient macrophages impede cardiac repair after myocardial infarction by enhancing the macrophage–myofibroblast transition

**DOI:** 10.1038/s12276-024-01304-0

**Published:** 2024-09-12

**Authors:** Hyang Hee Cho, Siyeon Rhee, Dong Im Cho, Ju Hee Jun, HyoJung Heo, Su Han Cho, Dohyup Kim, Mingqiang Wang, Bo Gyeong Kang, Soo Ji Yoo, Meeyoung Cho, Soo yeon Lim, Jae Yeong Cho, In Seok Jeong, Yong Sook Kim, Youngkeun Ahn

**Affiliations:** 1 Cell Regeneration Research Center, Chonnam National University Hospital, Gwangju, Republic of Korea; 2 Stanford Cardiovascular Institute, Stanford University, Stanford, CA USA; 3 Department of Molecular and Cell Biology, University of California, Berkeley, CA USA; 4 Department of Biology, Kyung Hee University, Seoul, Republic of Korea; 5 Division of Asthma Research, Cincinnati Children’s Hospital Medical Center, Cincinnati, OH USA; 6 Department of Cardiology, Chonnam National University Hospital and Medical School, Gwangju, Republic of Korea; 7 Department of Thoracic and Cardiovascular Surgery, Chonnam National University Hospital and Medical School, Gwangju, Republic of Korea; 8 Biomedical Research Institute, Chonnam National University Hospital, Gwangju, Republic of Korea

**Keywords:** Cell biology, Immunology

## Abstract

The regulatory role of the inhibitor of NF-kB kinase ε (IKKε) in postmyocardial infarction (MI) inflammation remains uncertain. Using an MI mouse model, we examined the cardiac outcomes of IKKε knockout (KO) mice and wild-type mice. We employed single-cell RNA sequencing (scRNA-seq) and phosphorylated protein array techniques to profile cardiac macrophages. IKKε KO mice exhibited compromised survival, heightened inflammation, pronounced cardiac fibrosis, and a reduced ejection fraction. A distinct cardiac macrophage subset in IKKε KO mice exhibited increased fibrotic marker expression and decreased phosphorylated p38 (p-p38) levels, indicating an enhanced macrophage–myofibroblast transition (MMT) post-MI. While cardiac inflammation is crucial for initiating compensatory pathways, the timely resolution of inflammation was impaired in the IKKε KO group, while the MMT in macrophages accelerated post-MI, leading to cardiac failure. Additionally, our study highlighted the potential of 5-azacytidine (5-Aza), known for its anti-inflammatory and cardioprotective effects, in restoring p-p38 levels in stimulated macrophages. The administration of 5-Aza significantly reduced the MMT in cardiac macrophages from the IKKε KO group. These findings underscore the regulation of the inflammatory response and macrophage transition by the IKKε-p38 axis, indicating that the MMT is a promising therapeutic target for ischemic heart disease.

## IKKε deficiency accelerates cardiac inflammation and fibrosis post-MI

Heart attacks can result in heart failure due to ongoing inflammation and scarring. This research investigates how a specific protein, IKKε, affects inflammation in heart cells. Researchers used genetically altered mice and sophisticated genetic methods to study IKKε‘s role in heart disease. They analyzed heart tissue after inducing heart attacks in the mice, focusing on how cells involved in inflammation and scarring behave differently when the IKKε protein is changed. Macrophage-myofibroblast transition (MMT) is a process in which macrophages acquire characteristics similar to myofibroblasts, potentially contributing to tissue fibrosis. The main discovery is that blocking IKKε causes more scarring by encouraging excessive MMT, suggesting it could be a target for heart disease treatment. The researchers believe that controlling the MMT process could be a new method to enhance heart health after a heart attack by reducing harmful scarring.

This summary was initially drafted using artificial intelligence, then revised and fact-checked by the author.

## Introduction

Myocardial infarction (MI) is a leading cause of heart failure globally, triggering chronic inflammation and cardiac fibrosis, which are common cardiac diseases. Uncontrolled inflammation hampers cardiac function and its crucial communication networks increases the risk of heart failure and increases the mortality rate post-MI. Patients who experience extensive myocardial injury during the acute phase of MI remain at risk of developing heart failure and continue to exhibit increased mortality rates. In the initial inflammation phase, immune cells such as macrophages actively participate in the clearance of cellular debris, induce angiogenesis, and promote proinflammatory signaling. Resident fibroblasts begin to proliferate and differentiate into myofibroblasts during the reparative phase, and macrophages are shifted toward the anti-inflammatory phenotype.

The nuclear factor-kappa B (NF-κB) family plays a pivotal role in the inflammatory signaling pathway, with complex involvement in the initiation, elongation, and termination of inflammation. For instance, activation of NF-κB in resident macrophages or leukocytes contributes to the resolution of inflammation^1,2^. NF-κB p65 has been shown to induce mechanisms for both the initiation and termination of the NF-κB pathway through NF-κB inhibitor α(IκBα) degradation and p65 phosphorylation, respectively^3^. Inhibitor of NF-κB kinase subunit epsilon (IKKε) is a noncanonical NF-κB member that has been identified as an inducible form in macrophages. IKKε has been reported to be induced in immune cells, especially in macrophages, by various stimuli, including inflammation^4,5^. Our main goal was to study the role of IKKε in macrophages in terms of the response to pathological inflammation in the infarcted heart. Thus, we investigated whether IKKε is involved in accelerating or resolving inflammation in macrophages in this study. In IKKε knockout (KO) mouse models, the levels of inflammatory cytokines are reduced^6^, and the lesion size is reduced in a high-fat diet-induced obesity model^7^, while cardiac hypertrophy increases in a pressure overload model^8^. Amlexanox, an inhibitor of IKKε and TANK-binding kinase 1 (TBK1), reduces inflammation and insulin resistance in obese mice^9^, prompting our exploration of IKKε in cardiac macrophages in an MI mouse model.

We examined the phenotypic diversity of macrophages after MI, and single-cell RNA sequencing (scRNA-seq) revealed a significant increase in the macrophage–myofibroblast transition (MMT) in IKKε KO mice with MI. During cardiac injury and repair, a cell state transition has been shown to play important roles in regulating injury and facilitating the repair process^10^. In various disease models, macrophages undergo a transition to produce fibrotic cells that contribute to physiological compensation. In a mouse renal fibrosis model, CD206(+) anti-inflammatory macrophages showed a greater rate of MMT than inflammatory macrophages^11^. In a chronic renal allograft injury model, interstitial fibrosis results from the transition of anti-inflammatory macrophages to myofibroblasts, which is regulated by a Smad3-dependent mechanism^12^. In addition to the renal fibrosis model, the cardiac MMT has been suggested for use in a myeloid cell-specific reporter mouse MI model^13^. Those authors showed that infiltrated macrophages expressed α-smooth muscle actin (αSMA), a myofibroblast marker, and fibroblast activation protein-α (FAP) at 1 week and collagen1α1 (COL1A1) and fibroblast-specific protein 1 (FSP1) at 6 weeks after MI. The cell state transition during tissue injury has been linked to the regulation of injury and repair processes. In various disease models, macrophages transition to fibrotic cells for physiological compensation. The MMT, a novel mechanism that alters macrophage function toward a fibrotic state, potentially plays a significant role in pathogenic cardiac fibrosis progression post-MI. Our findings support the occurrence of the MMT in infarcted myocardium and indicate that 5-azacytidine (5-Aza) effectively modulates the aberrant MMT in IKKε KO mice, suppressing pathological progression post-MI. This finding suggests a promising therapeutic approach for managing cardiac fibrosis and improving post-MI outcomes.

## Materials and methods

### Cell lines

The RAW264.7 murine monocyte/macrophage line, which was purchased from the Korean Cell Line Bank (Seoul, Korea), was cultured in Dulbecco’s modified Eagle’s medium (Gibco, 11885-084) supplemented with 10% fetal bovine serum (FBS) (Gibco, 16000044). THP-1 cells are a human monocyte cell line established from the peripheral blood of acute monocytic leukemia patients. THP-1 cells, purchased from the Korean Cell Line Bank, were cultured in RPMI-1640 (Welgene, LM-011-01) supplemented with 10% FBS and 1% penicillin‒streptomycin (PS) (Gibco, 15140-122). THP-1 cells were treated with 250 nM phorbol 12-myristate 13-acetate (PMA) (Sigma‒Aldrich, 8139) for 72 h to differentiate the THP-1 cells into macrophages.

### Mice and myocardial infarction model

Mice were bred and maintained under pathogen-free conditions at the Chonnam National University Medical School animal facilities. IKKε global KO mutant mice were purchased from the Jackson Laboratory (USA), and C57BL/6 J mice were purchased from OrientBio (Korea). All experiments were performed after approval by our local ethics committee at Chonnam National University Hospital (IRB No. CNU IACUC-H-2020-11). Male C57BL/6 J and IKKε KO mice (8 weeks of age) were anesthetized with an intramuscular injection of 50 mg/kg ketamine and 5 mg/kg xylazine, intubated, and mechanically ventilated. The proximal left anterior descending coronary artery was permanently ligated. Finally, the heart was repositioned in the chest, and the chest was closed. A total of 5 μg/g 5-Aza was preinjected into the peritoneal cavity one day before the induction of MI and was then injected every 2 days.

### Evaluation of cardiac function

Left ventricular (LV) function was assessed using echocardiography. At 2 weeks after MI induction, the mice were anesthetized, and echocardiography was performed with 15-MHz linear array transducer systems (iE33 system, Philips Medical Systems). The interventricular septal thickness (IVS), LV internal dimension (LVID), and LV posterior wall thickness (LVPW) at diastole and systole (IVSd, LVIDd, LVPWd, and IVSs, LVIDs, and LVPWs, respectively) were measured for echocardiographic analyses. The end-diastolic volume, end-systolic volume, LV ejection fraction (EF), LV fractional shortening (FS), and stroke volume were also measured. The echocardiography data were analyzed by an expert who was blinded to the genotype and experimental conditions.

### Histology

For histological studies, heart tissues were harvested, fixed with formalin, and embedded in paraffin blocks. The sections were cut at 4 μm for Masson’s trichrome staining and immunofluorescence analysis. Cardiac fibrosis was measured 7 days after MI using Trichrome Stain Kits according to the manufacturer’s protocol (Abcam, ab150686). Fibrotic areas were measured by visualizing blue-stained fibrosis using the NIS-Elements Advanced Research program (Nikon, Japan). The percentage of fibrosis was calculated as the blue-stained area of the left ventricle divided by the total area of the left ventricle. For the immunofluorescence analysis, the sections were permeabilized with 0.1% Triton X-100, and antigen retrieval was performed by boiling sections in 10 mM sodium citrate buffer. After the nonspecific binding was blocked with a 5% normal goat serum blocking solution (VECTOR, S1000), the sections were incubated with primary antibodies at 4 °C overnight. The sections were washed with PBS and then incubated for 1 h with Alexa Fluor 488-, Alexa Fluor 594-, or Cyanine5-conjugated secondary antibodies (Invitrogen). After washing, the sections were mounted with Gold antifade mountant with DAPI (Invitrogen, P36935). The images were detected using a Carl Zeiss confocal microscope, and the images were obtained using Zeiss LSM version 3.2 SP2 software (Carl Zeiss). The antibodies used are listed in Supplementary Table 4.

### Single-cell RNA sequencing

MI was induced in male C57BL/6 J and IKKε KO mice (8 weeks of age) for 4 days. Harvested hearts were washed with cold PBS and minced in a mixed enzyme solution containing 100 U/mL collagenase type 2 (Worthington Laboratories, LS004176) and 0.1% trypsin (Gibco, 15090046) in Hank’s balanced salt solution (HBSS) (Gibco, 14020-092) at 37 °C and 85 rpm for 5 min. The supernatant was discarded, a new mixed enzyme solution was added, and then the tissues were digested in the mixed enzyme solution at 37 °C and 85 rpm until the tissues disappeared. The supernatant was collected in cold culture medium composed of high-glucose Dulbecco’s modified Eagle’s medium (Welgene, LM 001-05) supplemented with 10% FBS and 1% PS. The samples were processed using 10x Genomics as a custom service by DNALINK (Korea).

### Single-cell RNA sequencing data analysis

The raw sequence data were mapped to the human reference genome GRCh38 using CellRanger v.7.1.0^14^ to create a count matrix for each sample. The Seurat R package v. 4.3.0 was used to cluster and analyze the cells from the count matrix^15^. Cells with fewer than 200 or more than 4500 transcripts or a 10% mitochondrial gene content were filtered out as low-quality cells. SCTransform v2^16^ was used to regularize and normalize the gene expression levels per sample. Uniform manifold approximation and projection (UMAP) was performed to reduce the dimensionality and visualize the gene expression data. The clusters were obtained using the FindNeighbors and FindClusters functions from the Seurat package. Each cluster was annotated to a unique cell type using both automated annotation software and manual curation^15,17^. For the RNA velocity analysis, the spliced and unspliced reads were counted using the velocyto package in R software (v0.17.17) from aligned bam files and barcodes generated by CellRanger and fed into velocyto to calculate RNA velocity values for each gene of each cell^18^. The resulting RNA velocity vector was embedded into the UMAP space.

### Isolation of bone marrow-derived macrophages (BMDMs)

Mononuclear cells were isolated from mouse bone marrow and cultured for 7–10 days in macrophage differentiation medium (supplemented with 30% L929 cell-conditioned medium, 20% FBS, and 50% RPMI-1640). RPMI-1640 was supplemented with 1% PS and 10% FBS. L929 cell-conditioned medium contained macrophage colony-stimulating factor secreted by L929 cells, which were cultured in RPMI-1640 supplemented with 10% FBS and 1% PS for 10 days. Finally, differentiated BMDMs were stimulated with 100 ng/mL lipopolysaccharide (LPS) (Sigma‒Aldrich, L4391), 30 ng/mL interferon γ (IFN-γ) (R&D Systems, 485-MI), 30 ng/mL IL-4 (R&D Systems, 404-ML), and 30 ng/mL IL-13 (R&D Systems, 413-ML).

### Isolation of cardiac cells for flow cytometry

Cardiac cells were isolated from mouse heart tissues according to a previously described method, with minor modifications^19^. Harvested hearts were minced and digested in a mixed enzyme solution containing 450 U/mL collagenase I (Sigma‒Aldrich, C0130), 125 U/mL collagenase XI (Sigma‒Aldrich, C7657), 60 U DNase I (Sigma‒Aldrich, DN25), and 60 U/mL hyaluronidase (Worthington Laboratories, 2592) in PBS supplemented with Ca^2+^/Mg^2+^ (Sigma‒Aldrich, D8662) for 1 h at 37 °C with gentle shaking. After the incubation, the digestion mixture was strained through a 40-μm nylon mesh. The collected cells were stained with phycoerythrin (PE)-conjugated antibodies and attached to anti-PE microbeads for magnetic cell sorting (MACS) to deplete undesired cells. Thy1.2 was excluded when examining the MMT. Cells were counted using a hemocytometer. Cell surface molecules were simultaneously stained with fluorophore-conjugated antibodies at room temperature for 30 min, and intracellular molecules were detected using a Fixation and Permeabilization Buffer Kit (R&D Systems, FC009). The antibodies used are listed in Supplementary Table 4. Cell fluorescence was measured with a BD FACSCanto II (BD Biosciences), and data were analyzed using FlowJo (BD).

### Isolation of macrophages and fibroblasts from mouse hearts

F4/80-positive cardiac macrophages were isolated from enzyme-digested hearts at 3 days post-MI using anti-F4/80 microbeads (Miltenyi Biotec, 130-110-443). Isolated cardiac macrophages were cultured in RPMI-1640 medium supplemented with 10% FBS and 1% PS and then stimulated with 100 ng/mL LPS, 30 ng/mL IFN-γ, 30 ng/mL IL-4, and 30 ng/mL IL-13. The MMT was induced in the cells by the addition of 5 ng/mL TGF-β1 (R&D Systems, 7666-MB) in RPMI-1640 supplemented with 1% FBS and 1% PS. The F4/80-negative cells were stained with a PE-conjugated Thy 1.2 antibody and attached to anti-PE microbeads for MACS. Isolated Thy 1.2-positive cardiac fibroblasts were cultured in high-glucose Dulbecco’s modified Eagle’s medium (WELGENE, LM 001-05) supplemented with 10% FBS and 1% PS. Secreted COL1A1 levels were evaluated using an enzyme-linked immunosorbent assay (ELISA) kit (Abcam, ab210579), and fibrotic genes were analyzed using real-time PCR.

### Isolation of human peripheral blood mononuclear cells

Human macrophages were differentiated from CD14-positive monocytes isolated from the peripheral blood of healthy donors. Blood sampling was performed under protocols approved by the Institutional Review Board of Chonnam National University Hwasun Hospital (IRB No. CNUHH-2021-045). Peripheral blood mononuclear cells (PBMCs) were isolated from peripheral blood by density centrifugation with Lymphoprep (STEMCELL, 07801) according to the manufacturer’s instructions. CD14-positive monocytes were separated from PBMCs using anti-CD14 mAb-coupled magnetic beads (CD14 MicroBeads; Miltenyi Biotec, 130-050-201) followed by MACS column separation according to the manufacturer’s protocol. Monocytes were seeded into tissue culture plates at a density of 3 × 10^5^ cells/mL for immunocytochemistry. Monocytes were treated for 5 days with 30 ng/mL human M-CSF (Sigma, M6518) in RPMI-1640 medium and differentiated into alternative macrophages for 3 days with 30 ng/mL M-CSF (Sigma‒Aldrich, M6518), 20 ng/mL IL-4 (R&D Systems, 204-IL), 20 ng/mL IL-6 (PeproTech, 200-06), and 20 ng/mL IL-13 (R&D Systems, 213-ILB). The macrophages were induced to undergo the MMT with 5 ng/mL TGF-β1 (R&D Systems, 7754-BH).

### Transfection of siRNAs

Small interfering RNAs, RNA-control (si-Con) and siRNA (si-*Ikbke*), were purchased from Bioneer (Korea). Cells were transfected with siRNAs using Lipofectamine RNAiMAX transfection reagent (Invitrogen, 13778150) according to the manufacturer’s protocols.

### Western blots

Protein from the cells and heart tissues was prepared in PRO-PREP Protein Extraction Solution (iNtRON, 17081) supplemented with a phosphatase inhibitor cocktail (Cell Signaling Technology, 5870). The cells and heart tissues homogenized in lysis buffer were incubated on a rotating wheel for 30 min at 4 °C. After centrifugation at 13,000 rpm for 15 min, the supernatant was prepared as a protein extract. Equal concentrations of proteins were fractionated by electrophoresis on 8%, 10%, or 12% acrylamide gels and transferred onto polyvinylidene fluoride membranes (Millipore, IPVH00010), followed by blotting with primary antibodies and secondary IgG antibody conjugated with horseradish peroxidase. Protein expression was detected using an image reader (MINI HD, UVITEC CAMBRIDGE). The antibodies used are listed in Supplementary Table 4.

### Phospho-antibody array

Wild type (WT) and IKKε KO BMDMs were treated with 100 ng/mL LPS and 30 ng/mL IFN-γ for 10 and 60 min. The cell lysates were applied to the Phospho Explore Antibody Array, which was designed and manufactured by Full Moon BioSystems and contains 1318 phospho-specific antibodies. These experiments and analyses were performed as a custom service by ebiogen (Korea).

### Immunocytochemistry

The cells were fixed with 4% paraformaldehyde for 15 min and washed three times with PBS. Then, the cell membrane was permeabilized with 0.1% Triton X-100 for 10 min, and antigen retrieval was performed by boiling the samples in 10 mM sodium citrate buffer. The samples were incubated with 5% normal animal serum in PBS for 1 h at room temperature, followed by staining with a primary antibody at 4 °C overnight. Subsequently, the cells were incubated with secondary antibodies conjugated with Alexa Fluor 488 (Molecular Probes, A11034) or Alexa Fluor 594 (Molecular Probes, A11037) for 1 h at room temperature, followed by mounting with Gold antifade mountant with DAPI (Invitrogen, P36935). The antibodies used are listed in Supplementary Table 4.

### Real-time PCR

The transcript levels were analyzed using real-time PCR. Total RNA was extracted with TRIzol reagent (Life Technologies, 15596018) and reverse transcribed to cDNA using an Applied Biosystems High-Capacity cDNA Reverse Transcription Kit (Life Technologies, 4368814) according to the manufacturer’s instructions. Real-time PCR was performed using a QuantiTect SYBR Green PCR kit (Qiagen, 204143) and a Corbett Research Rotor-Gene RG-3000 Real-Time PCR System. The expression levels of *GAPDH* and *ACTB* were used for normalization to calculate the expression levels of each gene. Human *CXCL9* and *CXCL10* were identified with predesigned primers from Bioneer (Korea). The primers used are listed in Supplementary Table 5.

### Collection of human heart tissue samples

Adult LV tissues were collected from heart transplant recipients at Chonnam National University Hospital (IRB No. CNUH-2023-006).

### Statistical analysis

All the data are presented as the means ± SEMs. Differences between the experimental and control groups were calculated using Student’s t-test or one-way ANOVA with Tukey’s multiple comparisons test with SPSS (SPSS Inc.). Significant *P* values are ^*^P < 0.05, ^**^P < 0.01, and ^***^P < 0.001.

## Results

### Cardiac injury is exacerbated in

#### IKKε knockout mice after MI

**Fig. 1:**
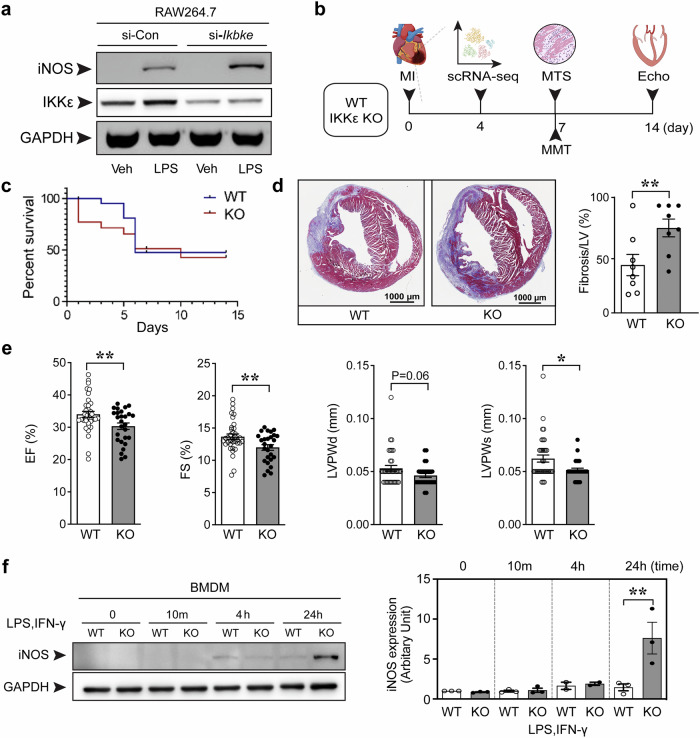
Cardiac injury and inflammation postmyocardial infarction in IKKε knockout mice. **a** RAW264.7 cells were transfected with the indicated siRNAs and stimulated with 100 ng/mL LPS for 4 h. **b** Schedule of the animal experiments. Adult (8 weeks) WT or IKKε KO mice were subjected to myocardial infarction (MI) or sham surgery, and hearts were harvested at the indicated times. Single-cell RNA sequencing (scRNA- seq), Masson’s trichrome staining (MTS), macrophage–myofibroblast transition (MMT) assays, and echocardiography (Echo) were performed. **c** The survival rates of the WT and IKKε KO mice after MI surgery. **d** Representative images and quantification of MTS to assess the scar size in mice 7 days after MI. Scale bars: 1000 μm. **e** Cardiac function was measured using echocardiography 14 days after MI. The ejection fraction (EF), fractional shortening (FS), left ventricular posterior wall thickness in diastole (LVPWd), and left ventricular posterior wall thickness in systole (LVPWs) were measured. **f** Bone marrow-derived macrophages (BMDMs) were isolated from WT and IKKε KO mice and cultured. Then, BMDMs were stimulated with 100 ng/mL LPS and 30 ng/mL IFN-γ for the indicated times. The induction of iNOS expression in BMDMs was assessed in WT and IKKε KO mice using Western blotting. The data are presented as the means ± SEMs. *P < 0.05 and **P < 0.01 (by Student’s *t-*test).

In acute injury, an inflammatory reaction inevitably involves the recruitment of immune cells to remove dead cells and cellular debris and to induce tissue repair. NF-κB is a master regulator of inflammation-related biological responses ranging from initiation to resolution. We transfected RAW264.7 cells with the IKKε siRNA before LPS stimulation to determine whether IKKε is involved in the inflammatory response in macrophages. IKKε knockdown was associated with increased inducible nitric oxide synthase (iNOS) expression (Fig. 1a), and we hypothesized that IKKε might be an endogenous inducer of inflammation resolution. We induced peritonitis in WT and IKKε KO mice to confirm the role of IKKε in macrophages under pathological conditions. Peritoneal macrophages were collected and showed higher expression of the inflammatory marker iNOS in IKKε KO mice than in WT mice (Supplementary Fig. 1a). Then, we investigated the role of IKKε in cardiac macrophages in a mouse model of MI. The experimental procedure is summarized in Fig. 1b. The survival rate was significantly lower (Fig. 1c). The histological analysis at 7 days post-MI revealed an increased fibrotic area (Fig. 1d), worsened cardiac function (Fig. 1e), and increased heart weight (Supplementary Fig. 1b) in IKKε-deficient mice compared with WT mice. Echocardiography was performed to determine LV function (Fig. 1e, Supplementary Table 1). The LV ejection fraction (EF) and LV fractional shortening (FS) were measured to evaluate LV global systolic function. The LVEF is the fraction of blood ejected from the LV during one heartbeat and is the parameter most commonly used as a surrogate of cardiac systolic function. By 7 days post-MI, LV systolic function was significantly diminished in the IKKε KO group, as reflected by an ~4% decrease in both the LVEF (WT 34.02% ± 5.53 vs. IKKε KO 30.33% ± 5.25, *p* < 0.01) and LVFS (WT 13.66% ± 2.51 vs. IKKε KO 11.99% ± 2.26, *p* < 0.01). Maintaining the LV posterior wall thickness (LVPW) contributes to contractile strength and ventricular structure, and the LVPW in systole (LVPS) was thinner in the IKKε KO group than in the WT group.

Representative molecules of the NF-κB pathway were compared between the two groups, and no difference was observed, except for IKKε, in heart tissues (Supplementary Fig. 1c). Importantly, IKKε KO BMDMs exhibited greater iNOS induction (Fig. 1f), consistent with the findings from IKKε-knockdown RAW264.7 cells (Fig. 1a). We isolated macrophages from the infarcted heart tissues to confirm the findings from BMDMs in heart tissues and found that the number of inflammatory cardiac macrophages was greater in the IKKε KO group than in the WT group (Supplementary Fig. 1d).

### Single-cell RNA sequencing reveals the characteristics of the macrophage–myofibroblast transition (MMT)

**Fig. 2:**
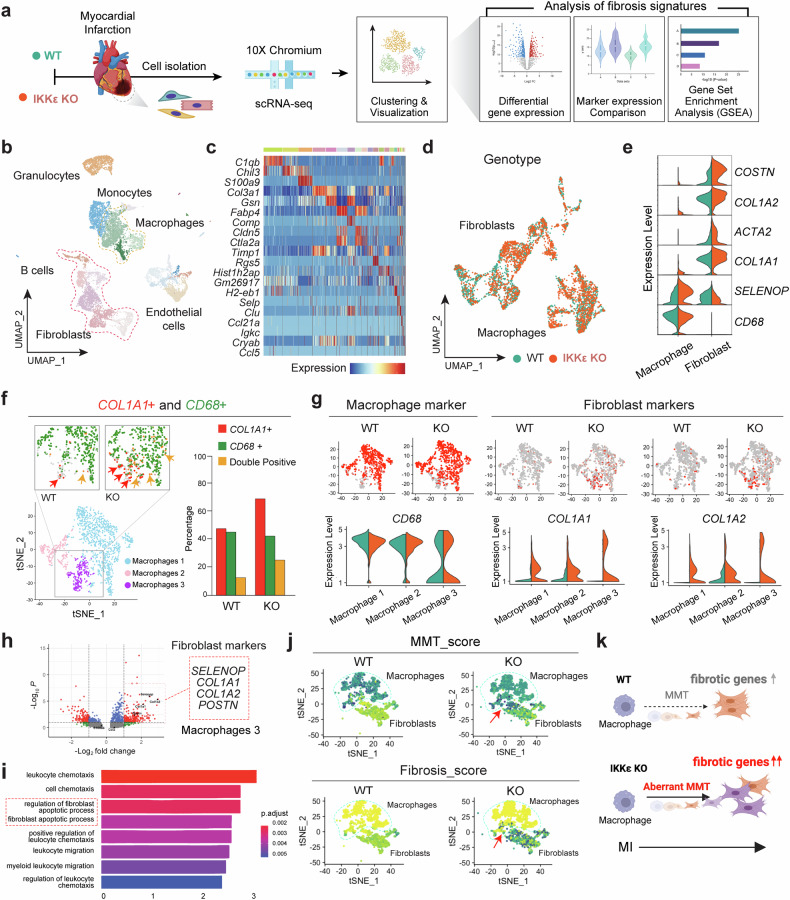
Single-cell RNA sequencing shows the proportion of macrophages expressing fibrotic markers. Cells were isolated from the infarcted myocardium for single-cell RNA sequencing. **a** Diagram of the experimental workflow for WT and IKKε KO mice postmyocardial infarction, followed by cell isolation, scRNA-seq, and a fibrosis analysis. **b** UMAP plot of cell clusters identified via scRNA-seq, including granulocytes, monocytes, macrophages, B cells, fibroblasts, and endothelial cells. **c** Heatmap displaying gene expression patterns across different cell types. **d** Genotype distinction in the UMAP plot showing WT and IKKε KO fibroblast and macrophage populations. **e** Violin plots showing fibrosis-associated gene expression levels in macrophages and fibroblasts. **f** t-SNE plots and quantitative analysis of *COL1A1* and *CD68* expression in WT and IKKε KO cells. **g** Comparison of the expression of macrophage and fibroblast markers between WT and IKKε KO samples via feature and violin plots. **h** Volcano plot showing the differential expression of fibroblast markers between WT and IKKε KO mice. **i** Gene Ontology enrichment analysis comparing the WT and IKKε KO transcriptomes. **j** t-SNE plots representing the differences in MMT scores between WT and IKKε KO cells. **k** A diagram showing the increased expression of fibrotic genes and aberrant MMT in IKKε KO mice postinfarction compared to those in WT mice.

Nonmyocyte cells were isolated from infarcted heart tissues (MI 4d) for the scRNA-seq analysis (Fig. 2a). Cells isolated from WT and IKKε KO mice were clustered, as suggested by Hao and Tao et al. (2021) (Supplementary Table 2). Cell type annotation was performed using automated packages and manual curation from representative genes in each cluster and visualized in a UMAP plot (Fig. 2b). All cell types identified through the scRNA-seq analysis in ventricular tissue are displayed on a heatmap along with their expression of representative marker genes (Fig. 2c). Additionally, after integrating the scRNA-seq data from two individual datasets, the UMAP plot showed that their distributions were well calibrated, indicating scalability for further analysis (Fig. 2d). The key genes of the fibrotic macrophage subsets were further visualized in a violin plot (Fig. 2e), and *COL1A1*(+)*CD68*(+) double-positive macrophages were quantified by comparing the WT and IKKε KO groups (Fig. 2f). We also validated that *COL1A1*(+)*CD68*(+) cells in the infarcted myocardium were found in a public spatial transcriptomics dataset (Supplementary Fig. 2a–d). Based on these data, we speculated that the MMT process occurs in the infarcted myocardium. The MMT is a cell transition from macrophages toward myofibroblasts and is usually determined by the coexpression of fibrotic and macrophage markers. One of the prominent differences was the upregulation of fibrotic markers in the macrophage subpopulation in the IKKε KO group (Macrophage 3) (Fig. 2g). We then performed a differential gene expression analysis and observed that the expression of fibrotic marker genes, such as *COL1A1, COL1A2, and POSTN* (Fig. 2h), and fibroblast regulation- and apoptosis-related gene sets were upregulated in Macrophage 3 in the IKKε KO group compared to the WT group (Fig. 2i).

We evaluated MMT (*CCR5, CD68, COL1A1*, and *COL1A2*) and fibrosis scores (*COL1A1* and *COL1A2*) using gene markers to investigate potential differences in the enrichment of MMT and fibrosis events between WT and IKKε KO mice (Fig. 2j). We replotted the MMT-positive macrophage subcluster (Cluster 3) identified from the subclustering analysis of the total macrophage population shown in Fig. 2f, g. This replotting enables a focused depiction of the expression profiles of pertinent genes/signatures within the MMT-positive macrophage subset on the tSNE plot. The same expression dataset was utilized for both Fig. 2d, j; however, Fig. 2d illustrates all macrophages and fibroblasts on a UMAP projection, whereas Fig. 2j is dedicated to showing the MMT-positive macrophage cluster on a tSNE plot. Our analysis revealed that the macrophage-to-mesenchymal and fibrosis signatures were more prevalent in IKKε KO mice than in WT mice, which is consistent with the experimental results (Fig. 2j). Based on the available data, we propose that the MMT process occurs in the infarcted myocardium. The MMT is a cellular transition in which macrophages transform into myofibroblasts, as indicated by the simultaneous expression of fibrotic and macrophage markers. A conceptual diagram illustrates that IKKε KO results in an increase in the expression of fibrotic genes and an aberrant MMT after MI (Fig. 2k). We narrowed the macrophage clusters and identified three specific macrophage clusters that are responsible for the MMT to obtain a better understanding of the pathophysiological significance of the MMT in infarcted hearts.

**Fig. 3:**
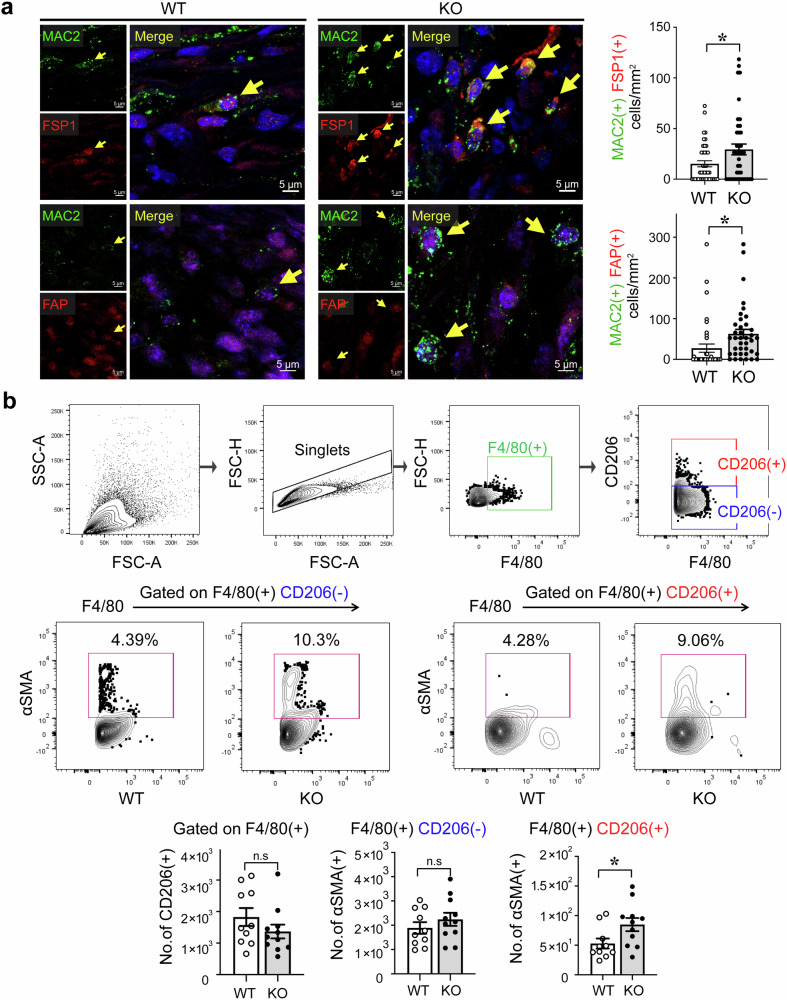
Fibrotic marker expression in macrophages in the infarcted myocardium. **a** Immunofluorescence staining showing the expression of fibrotic markers, including FSP1 and FAP, in MAC2(+) macrophages in the hearts of WT mice and IKKε KO mice at 7 days post myocardial infarction (MI). The number of double-positive cells was measured and is shown in the graph. Scale bars: 5 μm. **b** Cells were isolated from heart tissues at 7 days post-MI, and αSMA expression in F4/80(+)CD206(−) and F4/80(+)CD206(+) macrophages was analyzed via flow cytometry. The data are presented as the means ± SEMs. *P < 0.05 (Student’s *t-*test).

When we studied the expression of fibrotic markers in macrophage populations using scRNA-seq analysis, we found that FSP1(+)MAC2(+) macrophages in the infarcted myocardium were more frequent in the IKKε KO group than in the WT group. The number of FAP(+)MAC2(+) macrophages was also greater in the IKKε KO group (Fig. 3a). Consistent with the results of immunofluorescence staining, flow cytometry analyses revealed significantly more αSMA(+)CD206(+) macrophages in the IKKε KO group than in the WT group (Fig. 3b). Overall, in the infarct zone of the IKKε KO mice, the proportion of MMT cells (αSMA(+) macrophages) was greater than that in the WT mice.

### Macrophages acquire fibrotic phenotypes under MI conditions

**Fig. 4:**
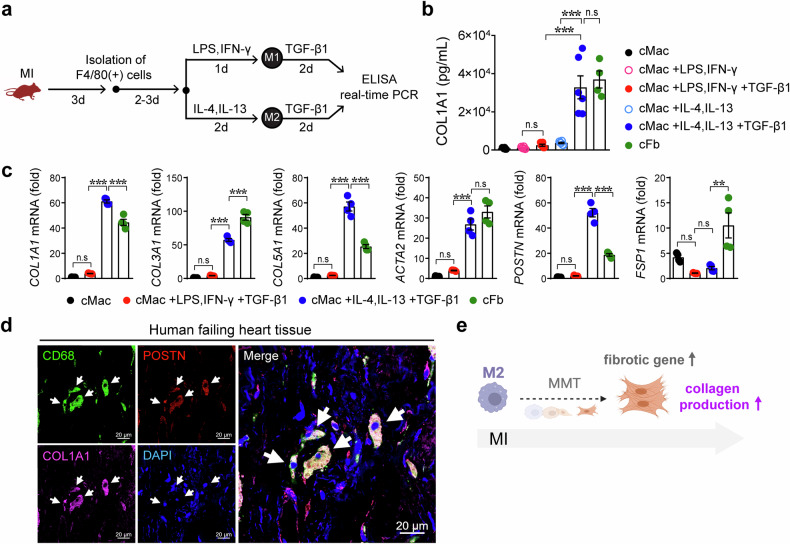
Macrophage–myofibroblast transition (MMT)-induced macrophages acquire fibrotic features after myocardial infarction. **a** Experimental protocol for inducing the MMT in cardiac macrophages (cMacs) isolated from WT mice with myocardial infarction (MI). cMacs were differentiated into inflammatory M1 macrophages with 100 ng/mL LPS and 30 ng/mL IFN-γ or into anti-inflammatory M2 macrophages with 30 ng/mL IL-4 and 30 ng/mL IL-13. Then, the cells were stimulated with the fibrosis inducer TGF-β1 (5 ng/mL) in culture media supplemented with 1% FBS. Isolated cardiac fibroblasts (cFbs) were used as a control group. **b** The amount of COL1A1 protein released in the culture media was measured. **c** Fibrotic genes were analyzed in cMacs and cFbs, as indicated. **d** Human heart tissues were isolated from consenting recipients with ischemic cardiomyopathy. Immunofluorescence staining revealed that periostin (POSTN)-expressing CD68(+) macrophages produced the collagen protein. White arrows, POSTN(+)CD68(+)COL1A1(+) cells. Scale bars: 20 μm. **e** Schematic representation showing that macrophages, particularly M2 macrophages, lead to the MMT and increased collagen production. The data are presented as the means ± SEMs. **P < 0.01 and ***P < 0.001 (one-way ANOVA with Tukey’s multiple comparisons test).

Next, we measured the level of the COL1A1 protein in the culture media to examine whether MMT-induced macrophages acquire fibrotic features (Fig. 4a). Cardiac macrophages (nonstimulated, M1, and M2) did not produce the COL1A1 protein, whereas the amount of COL1A1 released from TGF-β1-treated M2 cardiac macrophages was as high as that released from cardiac fibroblasts (Fig. 4b). The levels of fibrotic genes in cardiac macrophages and fibroblasts were compared, revealing the upregulation of fibrotic genes in MMT-induced anti-inflammatory cardiac macrophages (Fig. 4c). We next studied the MMT in human heart tissues. POSTN(+)CD68(+)COL1A1(+) cells were clearly observed in human tissues from patients with heart failure, indicating the presence of the MMT in human hearts (Fig. 4d). These results indicate that IKKε deficiency leads to the development of fibrosis by increasing the frequency of the MMT, particularly in M2 macrophages (Fig. 4e).

### IKKε knockout macrophages have low p38 activity

**Fig. 5:**
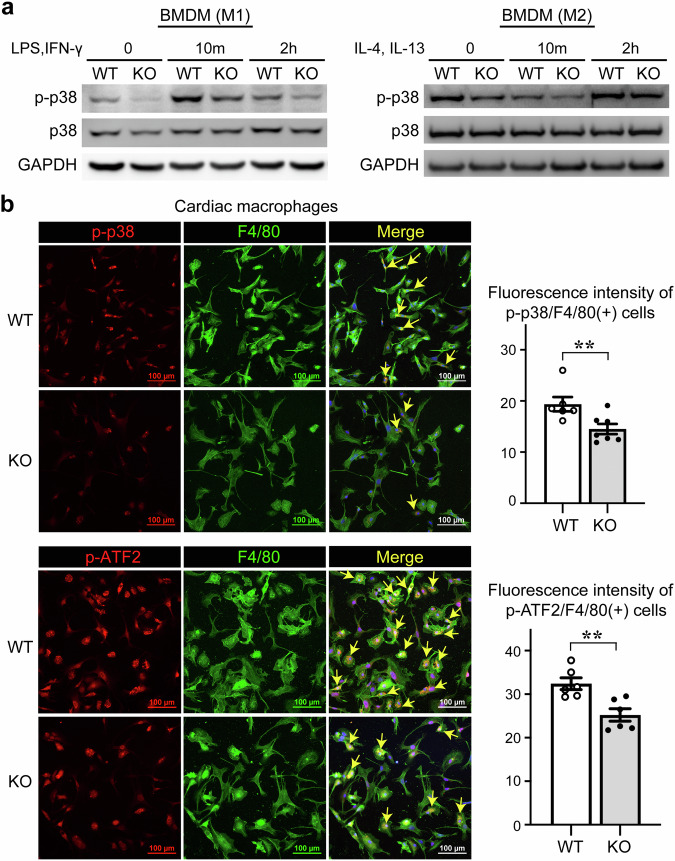
IKKε knockout mice have lower levels of phosphorylated p38 in macrophages. **a** Bone marrow-derived macrophages (BMDMs) were stimulated with 100 ng/mL LPS, 30 ng/mL IFN-γ, 30 ng/mL IL-4, and 30 ng/mL IL-13 for the indicated times. The protein levels of p-p38 and p38 in BMDMs were assessed in WT and IKKε KO mice using Western blotting. **b** Cardiac macrophages were isolated from heart tissues 3 days after myocardial infarction and immunofluorescence staining revealed the phosphorylation of p38 and its substrate ATF2. The fluorescence intensity indicating the phosphorylation of p38 and ATF2 was quantified in both the WT and IKKε KO groups. Scale bars: 100 μm. The data are presented as the means ± SEMs. **P < 0.01 (Student’s *t*-test).

We performed a phosphorylated protein array using BMDMs to identify the mediators responsible for the enhanced inflammation in IKKε KO macrophages. The level of phosphorylated p38 (p-p38) in IKKε KO BMDMs was lower than that in WT BMDMs when the BMDMs exhibited either the M1 phenotype or the M2 phenotype (Fig. 5a, Supplementary Table 3). We analyzed the protein levels of p-p38 and IKKε using Western blotting to explore the expression patterns of p-p38 and IKKε in the heart after MI over time. High p-p38 expression was detected as early as 1 day after MI and then gradually decreased after 3 days. The IKKε protein was barely expressed in the heart tissue of mice under normal conditions. However, its expression was induced and sustained 2 days after MI and then significantly decreased by 2 weeks (Supplementary Fig. 3a). In addition, using the same dataset as in Supplementary Fig. 2, we analyzed the longitudinal changes in *Ikbke* gene expression from Day 0 to Day 7 after MI. We observed an increase in *Ikbke* expression in the infarct zone (IZ) and border zone (BZ) following MI (Supplementary Fig. 3b). The restoration of p-p38 levels was delayed in the IKKε KO group 14 days after MI (Supplementary Fig. 3c). In addition to ischemic cardiac injury, we compared p-p38 levels in an angiotensin II-induced injury model and found that the p-p38 level in heart tissues was not restored in the IKKε KO group (Supplementary Fig. 3d). In human heart tissues, we also clearly noted the downregulation of p-p38 in the infarct zone (Supplementary Fig. 3e). Immunocytochemical staining also revealed that the levels of p-p38 and the phosphorylation of its substrate ATF2 were lower in cardiac macrophages isolated from IKKε KO mice than in those isolated from WT mice (Fig. 5b).

**Fig. 6:**
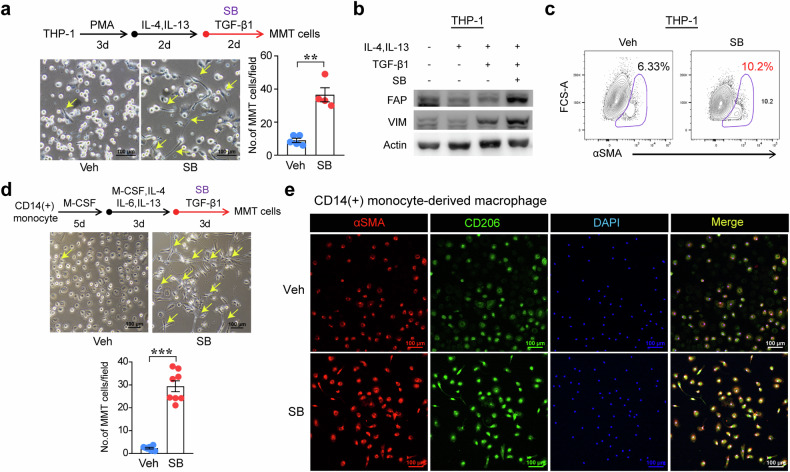
Active p38 in macrophages is involved in the macrophage–myofibroblast transition (MMT). **a** Experimental protocol used to induce the MMT in THP-1 cells. Monocytic THP-1 cells were differentiated into macrophages by treatment with 250 nM phorbol 12-myristate 13-acetate (PMA). Then, the cells were differentiated into an anti-inflammatory phenotype by treatment with 30 ng/mL IL-4 and 30 ng/mL IL-13. Next, 5 ng/mL TGF-β1 was added to induce the MMT, and during this process, the p38 inhibitor SB203580 (SB), was added to the culture media containing 1% FBS at a concentration of 10 μM. Representative images of cell morphology after MMT induction. The number of MMT cells was quantified by counting the number of cells with morphological changes. Scale bars: 100 μm. **b** The expression of fibrotic proteins, including FAP and vimentin (VIM), was assessed using Western blotting. **c** αSMA expression in MMT-induced THP-1 cells was assessed using flow cytometry. **d** The experimental protocol used to induce the MMT in human CD14(+) monocyte-derived macrophages treated with or without SB is shown. Cells were treated with 30 ng/mL M-CSF, 20 ng/mL IL-4, 20 ng/mL IL-6, and 20 ng/mL IL-13. A representative image of cell morphology is shown, and cellular changes were quantified. Scale bars: 100 μm. **e** αSMA expression in CD206(+) human anti-inflammatory macrophages, as shown by immunofluorescence staining. Scale bars: 100 μm. The data are presented as the means ± SEMs. **P < 0.01 and ***P < 0.001 (Student’s *t-*test).

Next, we examined whether p38 activity affects the macrophage phenotype using the p38 inhibitor SB203580 (SB). In LPS-stimulated RAW264.7 cells, iNOS induction was enhanced by treatment with SB (Supplementary Fig. 4a). In THP-1-derived macrophages, treatment with SB also upregulated inflammatory markers, including *CXCL9* and *CXCL10*, after LPS and IFN-γ stimulation (Supplementary Fig. 4b). Then, we switched THP-1-derived macrophages to the M2 phenotype by treating the cells with IL-4 and IL-13, and the fibrotic environment was recapitulated by TGF-β1 treatment to induce MMT with αSMA expression (Supplementary Fig. 4c). The MMT-induced morphological changes and cells tended to exhibit an elongated spindle-shaped morphology in the presence of SB (Fig. 6a). In addition, the induction of fibrotic markers such as FAP, Vimentin, and αSMA was augmented in MMT-induced THP-1 cells by SB treatment (Fig. 6b, c). We differentiated CD14(+) monocytes from peripheral blood into M2-type macrophages with M-CSF and interleukin treatments following TGF-β1 treatment with or without SB to validate the MMT process in primary human cells. Following treatment with SB, the morphology of the MMT-induced cells was significantly altered, and their numbers increased (Fig. 6d). Additionally, the expression of αSMA (Fig. 6e) and Vimentin (Supplementary Fig. 4d) was augmented in the SB-treated group. In cardiac macrophages isolated from WT mice on Day 3 after MI, the induction of the fibrotic mediator FSP1 was strongly enhanced by cotreatment with TGF-β1 and SB (Supplementary Fig. 4e).

### Control of MMT with 5-azacytidine (5-Aza) is associated with cardiac repair

**Fig. 7:**
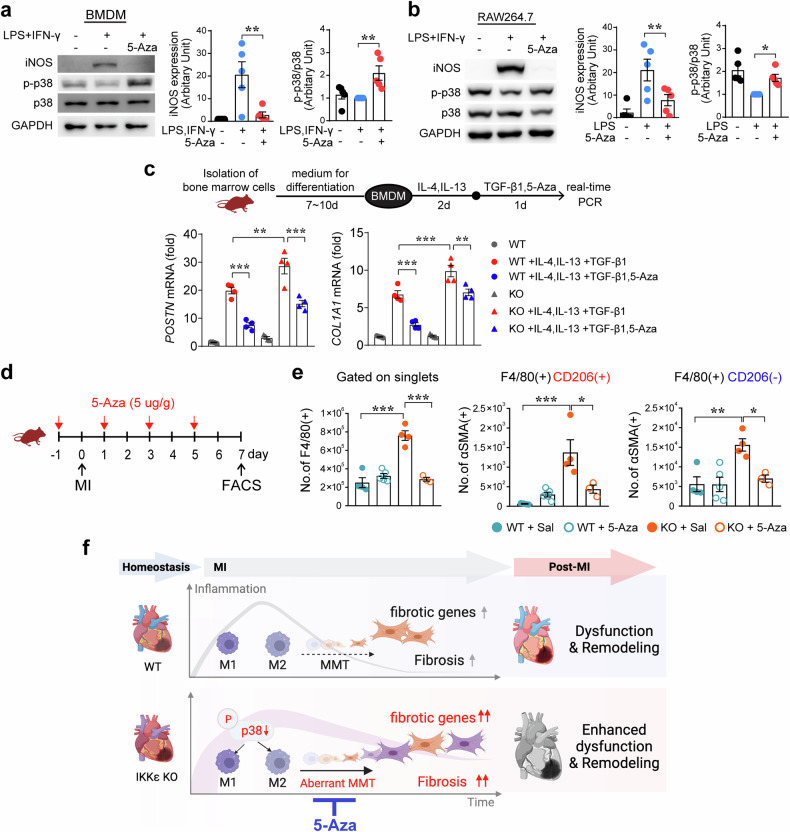
Control of the MMT by 5-Aza through the restoration of phosphorylated p38 levels in macrophages. **a**, **b** Stimulated bone marrow-derived macrophages (BMDMs) and RAW264.7 cells were treated with 10 μM 5-azacytidine (5-Aza) to examine the involvement of p-p38 activity in anti-inflammatory activity. The protein levels of iNOS, p-p38, and total p38 were assessed by Western blotting. **c** Bone marrow cells isolated from WT and IKKε KO mice were differentiated into BMDMs and then treated with 30 ng/mL IL-4 and 30 ng/mL IL-13, followed by treatment with 5 ng/mL TGF-β1 and 10 μM 5-Aza. Finally, *POSTN* and *COL1A1* expression were analyzed using real-time PCR. **d** Experimental protocol used to examine the effect of 5-Aza on MMT induction. Saline or 5-Aza was administered to mice with myocardial infarction (MI), and cardiac macrophages were isolated for flow cytometry analysis to quantify the MMT. **e** Cardiac cells were isolated from mice, and the phenotypes of cardiac macrophages were compared among the following groups: WT+saline, WT+5-Aza, IKKε KO+saline, and IKKε KO+5-Aza. **f** Schematic illustrations showing that the MMT was strongly induced in IKKε-deficient macrophages with low p38 activity. An aberrant MMT was associated with enhanced dysfunction and remodeling after MI. The data are presented as the means ± SEMs. *P < 0.05, **P < 0.01, and ***P < 0.001 (one-way ANOVA with Tukey’s multiple comparisons test).

We previously reported the anti-inflammatory effect of 5-Aza on macrophages through IRF-1 sumoylation, and we showed that administration of 5-Aza attenuated cardiac injury in an MI rat model^20,21^. We examined whether 5-Aza modulated p-p38 levels in macrophages. LPS-stimulated BMDMs and RAW264.7 cells were treated with 5-Aza, and we found that the level of p-p38 was significantly restored by 5-Aza treatment and that iNOS expression was suppressed (Fig. 7a, b). Then, we isolated BMDMs to study the effect of 5-Aza on the MMT in WT BMDMs and IKKε KO BMDMs. The increased levels of *POSTN* and *COL1A1* were downregulated by 5-Aza treatment in both the WT group and the IKKε KO group. Overall, the levels of *POSTN* and *COL1A1* were higher in the IKKε KO group than in the WT group. However, the levels of these genes in the IKKε KO group were reasonably decreased compared with those in the WT group following 5-Aza treatment (Fig. 7c). We administered 5-Aza to a mouse MI model to investigate whether 5-Aza affects the MMT in infarcted hearts. 5-Aza or saline was injected into mice with MI, and cardiac macrophages were isolated to compare the cell phenotypes using flow cytometry (Fig. 7d, e and Supplementary Fig. 5). The number of F4/80(+) macrophages was greater in the IKKε KO group than in the other groups, whereas the number of F4/80(+) macrophages was lower in the 5-Aza-treated IKKε KO group. Furthermore, both F4/80(+)CD206(+) macrophages and F4/80(+)CD206(−) macrophages in the 5-Aza-injected groups contained fewer αSMA(+) cells than in the control group (Fig. 7e). These data indicated that the increase in MMT activity in the IKKε KO group significantly returned to the level detected in the WT group at 7 days post-MI following 5-Aza administration. We performed additional animal experiments to investigate the effects of 5-Aza on infarcted hearts. We compared the distribution of POSTN(+) fibroblasts within the infarcted myocardial tissues and found that the fibroblast distribution was greater in the IKKε KO group than in the WT group. However, 5-Aza administration significantly reduced the fibroblast distribution in both groups (Supplementary Fig. 6a). After MI, the worsened alignment of the gap junction protein Connexin43 between cardiac Troponin I(+) cardiomyocytes and impaired angiogenesis in the IKKε KO group were improved in both groups by 5-Aza administration (Supplementary Fig. 6b).

Taken together, our findings revealed that IKKε KO macrophages have lower p-p38 levels, resulting in an aberrant MMT during MI and that an enhanced MMT is implicated in adverse outcomes, such as severe fibrosis and cardiac dysfunction (Fig. 7f).

## Discussion

Ischemic damage elicits repair responses that promote the removal of cell debris, the recruitment of immune cells, and the initiation of angiogenesis. Macrophages play a central role in the reparative response. Following heart damage, macrophages actively participate in clearing cell debris and activating inflammatory signaling to promote angiogenesis. In the reparative phase, macrophages are shifted toward anti-inflammatory actions to induce stable scar formation. Timely activation of macrophages is crucial for compensatory and reparative processes. However, excessive inflammation results in adverse remodeling with cardiac dysfunction. Inflammation and adverse outcomes are known to be linked in cardiovascular disease. However, the results of clinical trials targeting cardiovascular inflammation have been disappointing. For instance, in the representative CANTOS trial of anti-inflammatory therapy, the monoclonal antibody canakinumab, which targets interleukin-1β (IL-1β), reduced the plasma levels of IL-6 and CRP and the endpoint of cardiovascular death. On the other hand, canakinumab has not entered the cardiovascular therapeutic arena because of the potential risk of fatal infection. The failure of anti-inflammatory therapy may be related to a lack of an in-depth understanding of the pathophysiological roles of inflammation dynamics in multiple phenotypes of immune cells.

IKKs consist of canonical components (IKKα, β, and γ) and an alternative kinase (IKKε), according to their structural features. IKKα is involved in the termination of NF-κB-dependent inflammatory signaling. RelA and c-Rel are phosphorylated by IKKα and then cleared from target gene promoters, resulting in the negative regulation of inflammatory macrophages. When IKKα function is impaired, IKKα fails to terminate macrophage activation, thus increasing local inflammation^22^. IKKβ suppresses the inflammatory macrophage phenotype to prevent the excessive activation of macrophages during inflammation^23^. IKKγ may exert a protective function in the parenchymal compartment during the pathogenesis of pancreatitis. In chronic pancreatitis, the deletion of IKKγ in epithelial cells aggravates inflammation and fibrosis and delays recovery^24^.

The role of IKKε is diverse in the disease context. In obese mice, IKKε deficiency prevents the initial macrophage inflammatory response to a high-fat diet^6^. In an aortic banding model, IKKε deficiency increases cardiac hypertrophy and fibrosis^13^. In ApoE KO mice, IKKε deficiency inhibits high-fat diet-induced inflammation and obesity^25^. High-fat diet-induced atherosclerosis in ApoE KO mice is reduced in IKKε KO mice^7^. Additionally, in ApoE KO mice, the ablation of IKKε enhances and prolongs NLRP3 inflammasome priming and metaflammation. IKKε-deficient BMDMs and peritoneal macrophages respond more strongly to LPS stimulation, and IKKε and TBK1 have been suggested to act as counterinflammatory kinases to attenuate the inflammatory response^26^. In terms of the seemingly incompatible results for the impact of IKKε on cardiovascular disease models, the outcomes seemed to be highly dependent on obesity. In other words, the findings suggest that IKKε may act as an inflammation brake in lean mouse models and as an inflammation accelerator in models of obesity. In this study, we used C57BL/6 mice fed standard chow, and IKKε KO macrophages elicited a greater inflammatory response than did WT macrophages (Fig. 1f). Similarly, LPS-induced iNOS induction was enhanced in IKKε-knockdown RAW264.7 cells (Fig. 1a). These initial results indicated that IKKε might be involved in inflammation resolution, and we analyzed the RNA transcription profiles of cardiac macrophages.

For the cellular dissection of inflammation-related features in cardiac macrophages during MI, clustering and integration analyses were performed using Seurat (Fig. 2a). Unsupervised clustering identified key cardiac cell types (Fig. 2b), each with distinct cluster-specific markers (Fig. 2c). We initially isolated fibroblast and macrophage subsets to characterize macrophages and fibroblasts post-MI. Although the proportion of *CD68*(+) macrophages remained similar, *COL1A1* expression and the percentage of *COL1A1*(+)*CD68*(+) cells increased in the IKKε KO group (Fig. 2e, f). The macrophages in the IKKε KO group expressed higher levels of fibrotic genes (Fig. 2g). This specific macrophage subpopulation was larger in the IKKε KO group than in the WT group, suggesting that this subpopulation was more frequent in the IKKε KO group (Fig. 2h). The differentially expressed genes in the Macrophage 3 cluster included fibrotic genes, such as *COL1A1* and *COL1A2*, for which the GO enrichment analysis revealed that the top genes are involved in fibroblast apoptosis and its regulation, which align with key signatures of the MMT. These findings led us to investigate the cell transition of macrophages toward fibrotic cells.

Then, we explored phosphorylated proteins to identify the substrates of IKKε and found that IKKε KO BMDMs and cardiac macrophages exhibited significantly lower levels of phosphorylated p38 than did WT cells. Furthermore, treatment of macrophages with the p38 inhibitor SB resulted in enhanced inflammatory responses. We compared the molecular signatures in nonmyocytes isolated from both IKKε KO mice and WT mice at 4 days after MI using scRNA-seq to analyze the features of enhanced inflammation in macrophages. scRNA-seq analyses indicated that macrophages acquire fibrotic gene signatures that result in phenotypic and functional changes and may lead to fibrosis. This unexpected finding led us to turn our attention to the cell transition.

Responses to cardiac injury involve dynamic interactions between individual cells that collectively regulate heart architecture and function. Cell populations are in continuous communication with their counterparts in response to injury signals. In the infarcted myocardium, macrophages proliferate and are dynamically recruited from the bone marrow and spleen, resulting in diverse phenotypes. Resident fibroblasts rapidly move to the infarcted lesion to become myofibroblasts, contributing to the wound-healing process. Myofibroblasts originate from a number of sources^27^, including epithelial cells^28,29^, endothelial cells^30^, fibroblasts, pericytes^30^, and bone marrow^31^. Upon acute injury, cell phenotypes are not stable; these cells can change their functions with mixed identities in their microenvironment. This phenomenon is known as a cell transition and is triggered in response to pathophysiological stimuli. Cell transitions, including the epithelial–mesenchymal transition (EMT), endothelial-to-mesenchymal transition (EndMT), and mesenchymal-to-endothelial transition (MEndoT), is the process by which cells change their state during embryonic development, homeostasis, and tissue repair. In terms of the fibrotic changes in the myocardium, the EndMT is induced by TGF-β1 to contribute to the accumulation of cardiac fibroblasts in a pressure overload mouse model, and the administration of BMP-7, a member of the TGF-β1 superfamily, significantly inhibits the EndMT and the progression of cardiac fibrosis by targeting fibroblasts carrying an endothelial imprint^30^.

The MMT is a complex process in which macrophages partially lose their phenotypic and functional characteristics and acquire myofibroblast-like features. The MMT was identified as the mechanism by which ongoing inflammation causes progressive fibrosis in diseased tissues. In the early stages of benign prostatic hyperplasia, CD68(+)CD163(+) anti-inflammatory macrophages are converted to the myofibroblast phenotype and promote stromal fibrotic tissue remodeling^32^. We observed the MMT in the infarcted myocardium of WT mice and more frequently in IKKε KO mice. More importantly, the collagen-producing MMT was detected in human failing heart tissues. To the best of our knowledge, this report is the first showing the MMT in human heart tissues. Although the mechanisms underlying the cardiac MMT are still largely unknown, the MMT may provide several advantages for the restoration of tissue structure and function. Namely, alternative types of macrophages may additively support the formation of a stable fibrotic scar to maintain cardiac pumping.

We have previously reported that 5-Aza exerts cardioprotective effects through anti-inflammatory activity on an angiotensin II-infusion cardiac infusion mouse model and a rat MI model^20,21^. After finding that 5-Aza treatment restored p-p38 levels in the stimulated macrophages, we examined whether the mechanism of 5-Aza might be related to normalizing the MMT. Interestingly, the MMT in the IKKε KO group was reduced by 5-Aza administration to a level similar to that in the WT group. These encouraging findings warrant further development of precision-targeted therapeutics for cardiovascular and fibrotic diseases.

The development of cardiovascular therapeutics has been limited by an incomplete understanding of the diverse phenotypes of macrophages and the origin of fibroblasts in the heart. Because the relative contribution of inflammation is likely dependent on contextual factors, we need to stratify cardiac pathophysiology, and we need to understand to what extent inflammation contributes to an appropriate MMT to identify therapeutic targets. Overall, our data suggest that the IKKε-p38 axis in macrophages may be involved in the regulation of the MMT. Additional studies using lineage tracing models and human cardiac tissues are needed to further elucidate the regulators of MMT. Macrophages are a heterogeneous population with an important role in heart homeostasis, and the modulation of the MMT may be a key signaling mechanism for cardiac therapeutics.

## Supplementary information

Supplementary information

Supplementary Table 2

Supplementary Table 3
