# Supplementary material for: IKKε-deficient macrophages impede cardiac repair after myocardial infarction by enhancing the macrophage–myofibroblast transition
Source: Exp Mol Med. 2024 Sep 12;56(9):2052–64. doi: 10.1038/s12276-024-01304-0 (PMC11446912; doi:10.1038/s12276-024-01304-0)
Supplement: Supplementary file 1 — Supplementary information [file 12276_2024_1304_MOESM1_ESM.pdf]

Supplementary information

IKKε-deficient macrophages impede cardiac repair after myocardial infarction by enhancing the macrophage–myofibroblast transition

Running title: Macrophage-Myofibroblast Transition in the Hearts

Hyang Hee Cho[1†], Siyeon Rhee[2†], Dong Im Cho[1], Ju Hee Jun[1], HyoJung Heo[3], Su Han Cho[4], Dohyup Kim[5], Mingqiang Wang[2], Bo Gyeong Kang[1], Soo Ji Yoo[1], Meeyoung Cho[1], Soo yeon Lim[1], Jae Yeong Cho[6], In Seok Jeong[7], Yong Sook Kim[1,8]*, and Youngkeun Ahn[1,6]*

[1]Cell Regeneration Research Center, Chonnam National University Hospital, Gwangju, Republic of Korea.

[2]Stanford Cardiovascular Institute, Stanford University, Stanford, California, USA.

[3]Department of Molecular and Cell Biology, University of California, Berkeley, California, USA.

[4]Department of Biology, Kyung Hee University, Seoul, Republic of Korea

[5]Division of Asthma Research, Cincinnati Children's Hospital Medical Center, Cincinnati, Ohio, USA.

[6]Department of Cardiology, Chonnam National University Hospital and Medical School, Gwangju, Republic of Korea.

[7]Department of Thoracic and Cardiovascular Surgery, Chonnam National University Hospital and Medical School, Gwangju, Republic of Korea

[8]Biomedical Research Institute, Chonnam National University Hospital, Gwangju, Republic of Korea.

[†] These authors contributed equally to this article.

*Address for correspondence:

Youngkeun Ahn, Department of Cardiology, Chonnam National University Hospital and Medical School, Gwangju, 61469, Republic of Korea.

Tel: 82-62-220-6188, Email: cecilyk@hanmail.net

Yong Sook Kim, Biomedical Research Institute, Chonnam National University Hospital, Gwangju, 61469, Republic of Korea.,

Tel: 82-62-220-6941, Email: reorgan@hanmail.net

Supplementary Fig 1.

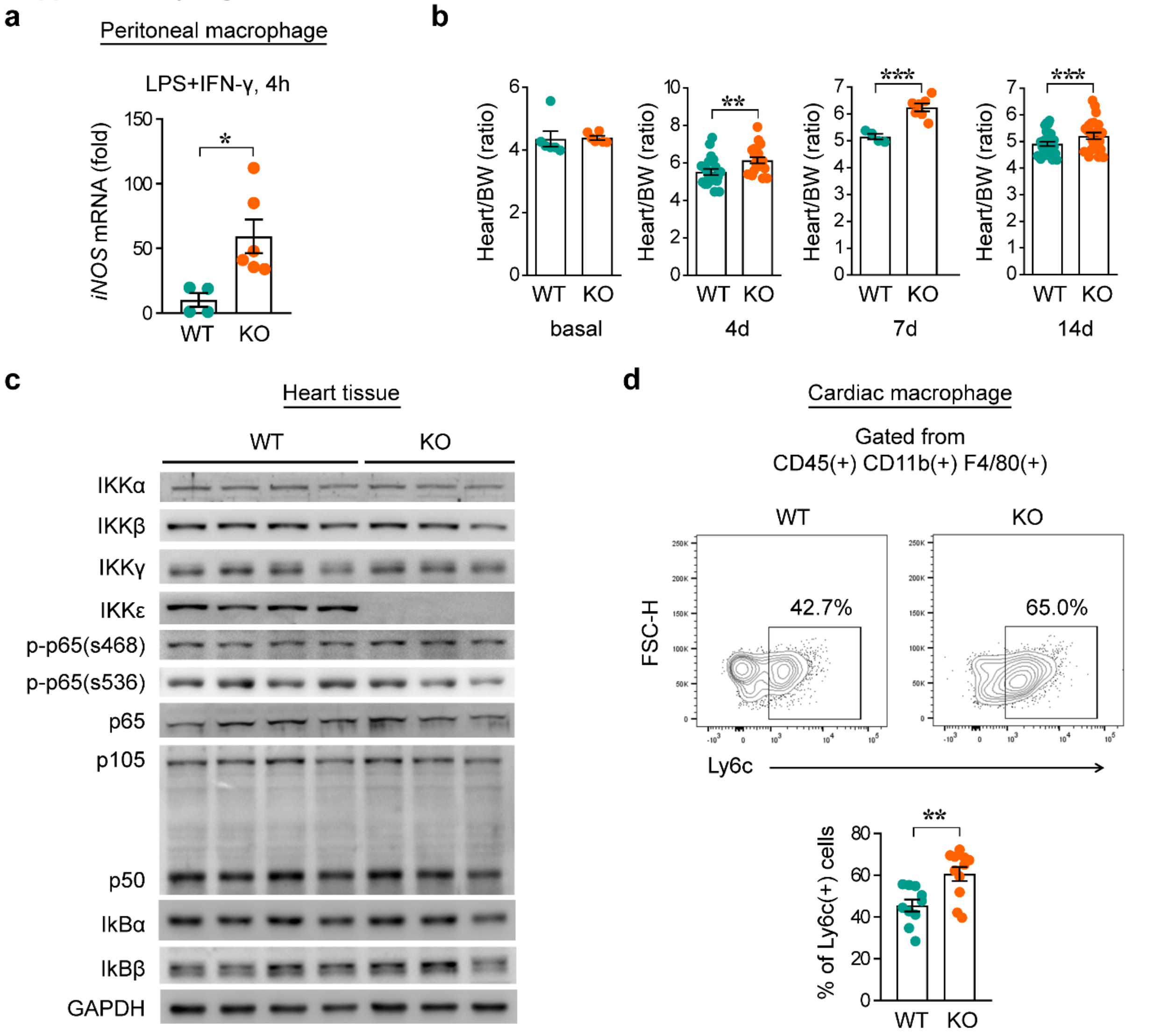

Supplementary Fig 1. IKKε deficiency is associated with enhanced cardiac damage after myocardial infarction. a Peritoneal macrophages were isolated from a peritonitis model in wild type (WT) and IKKε knockout (KO) mice, and stimulated 100 ng/mL LPS and 30 ng/mL IFN-γ for 4 hours. The *iNOS* mRNA level was assessed by real-time PCR. **b** Hearts were collected at day 0, 4, 7, and 14 after myocardial infarction (MI), and the ratio of heart to body weight was measured. **c** The protein levels of four types of IKK and NF-κB subtypes were compared in WT and IKKε KO mice after for 4 days of MI. **d** Cells were isolated from infarcted heart tissues to compare the

number of inflammatory macrophages. Data are represented as mean ± SEM. *$P < 0.05$; **$P < 0.01$; ***$P < 0.001$ (by Student's t test).

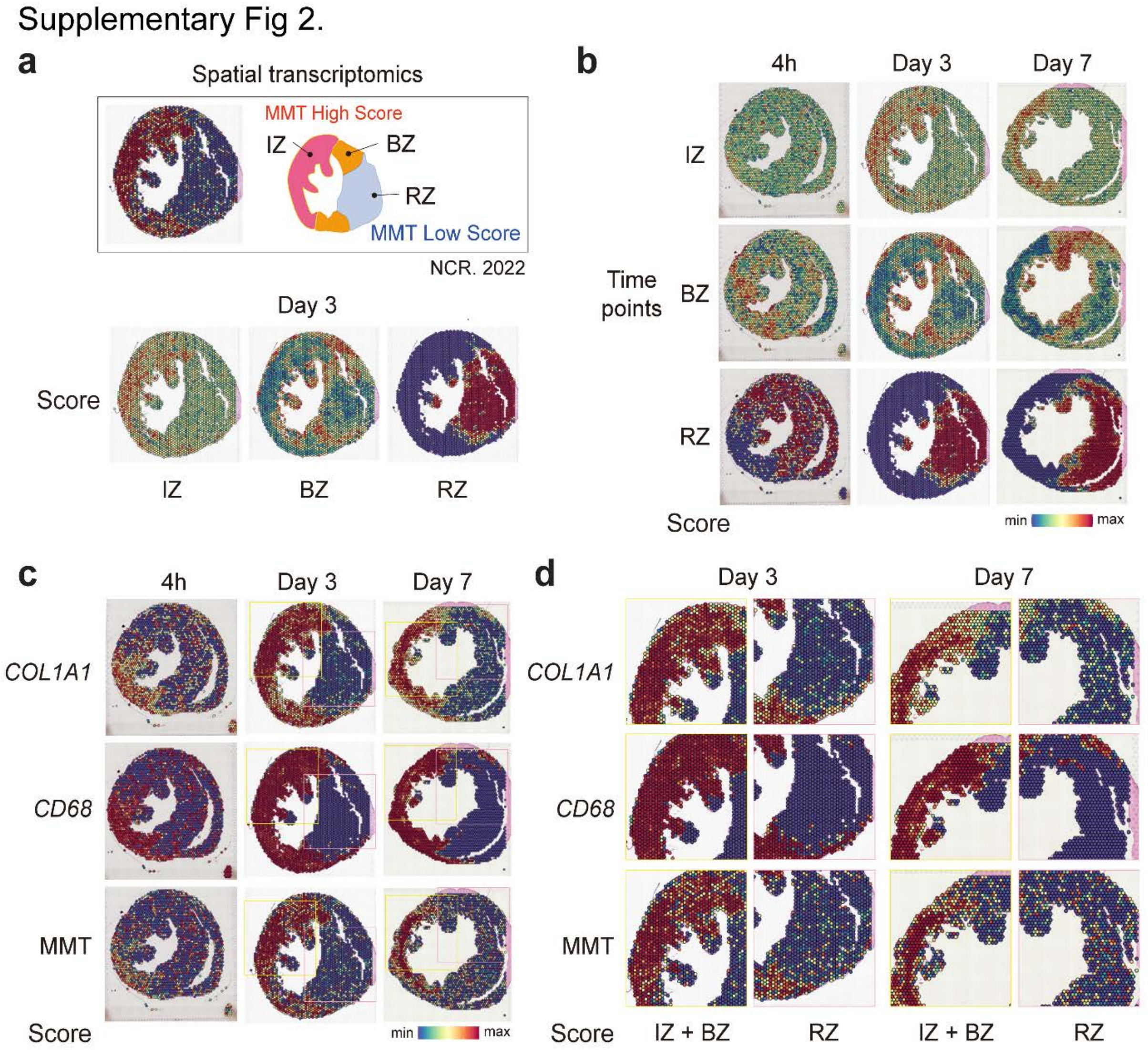

Supplementary Fig 2. The portions of fibroblasts, macrophages, and MMT were visualized with integration of myocardial infarction spatial dataset. **a** Overview of spatial transcriptomic analysis across infarct zones. The top-left image depicts the high and low infarct scores in different zones: Infarct Zone (IZ), Border Zone (BZ), and Remote Zone (RZ). These zones are defined by marker genes as previously reported by Calcagno et al. (Nature Cardiovascular Research, 2022). Below, a time series illustrates the spatial distribution of gene expression scores on day 3 post-myocardial infarction across the IZ, BZ, and RZ. **b** Time course representation of spatial gene

expression within IZ, BZ, and RZ at 4 hours, day 3, and day 7 post-MI, demonstrating the dynamic changes in the transcriptomic landscape. The figure illustrates that border marker genes' expression patterns progressively increase over time after myocardial infarction. **c** Detailed spatial transcriptomics for selected genes (*COL1A1*, *CD68*, and *CD68+COL1A1+*) at 4 hours, day 3, and day 7 post-MI, providing insights into the cellular and molecular changes within the cardiac tissue during the healing process. Also, the figure illustrates that the expression patterns of our selected genes, along with the MMT score, progressively increase over time. **d** High-resolution analysis of *COL1A1* and *CD68* gene expression along with MMT scores, focusing on the IZ+BZ and RZ at days 3 and 7 post-MI. This analysis highlights the differential gene expression patterns between the infarct and remote zones. Compared to RZ, expression score of our selected genes (*COL1A1*, *CD68*), along with MMT score is higher, which implies the process of MMT in infarcted myocardium.

Supplementary Fig 3.

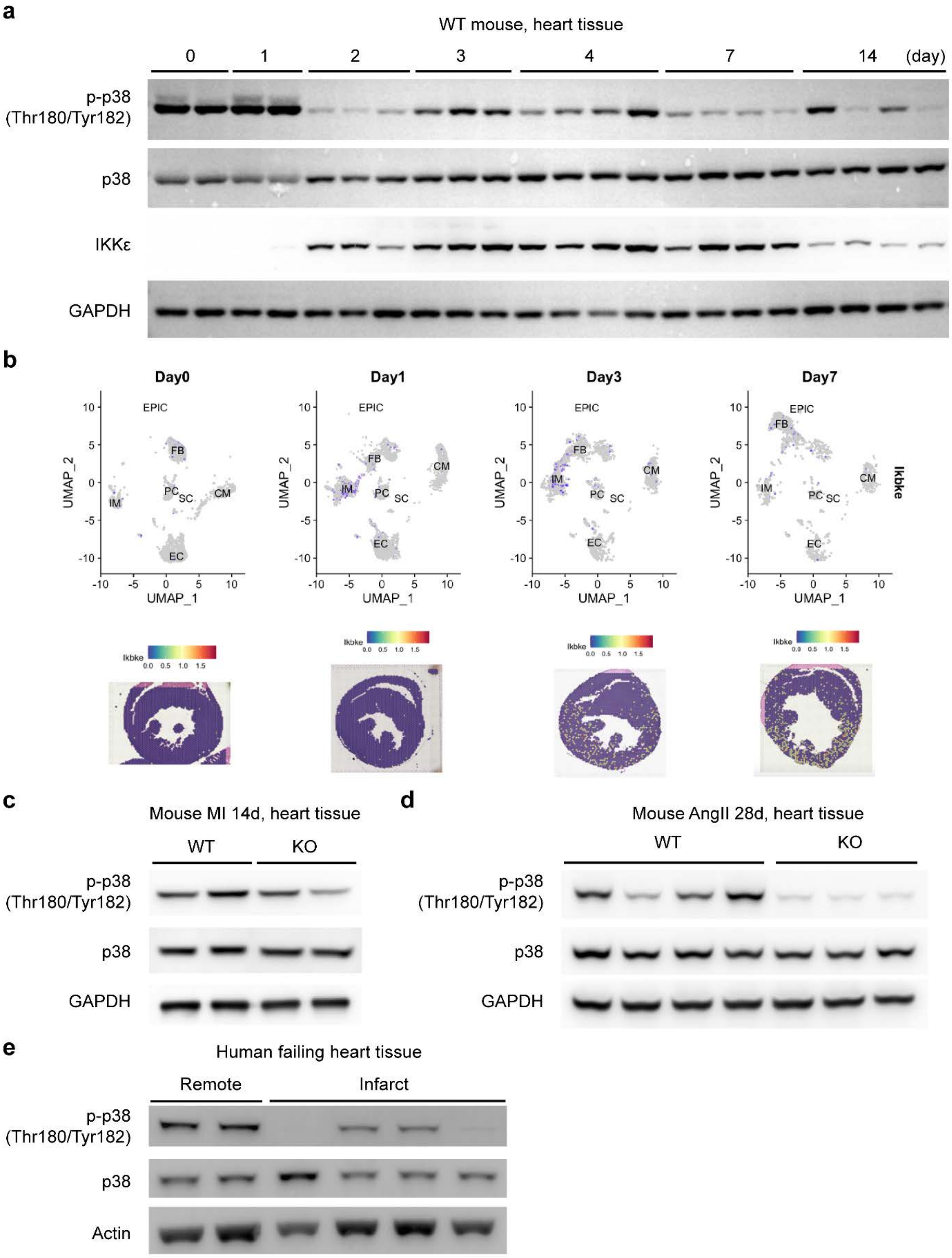

Supplementary Fig 3. Phosphorylated p38 in damaged heart tissues. a Mouse hearts were collected at day 0, 1, 2, 3, 7, and 14 after myocardial infarction (MI) to assess the protein levels of phosphorylated p38 (p-p38) and IKKε by Western blot. **b** Subanalysis of the dataset in Supplementary Fig 2 showed that the expression of the *Ikbke* gene significantly increased from day 3 to 7 after MI. **c** Protein levels of p-p38 in heart tissues of wild type (WT) and IKKε knockout (KO) mice after 14 days of MI. **d** Protein levels of p-p38 in heart tissues of WT and IKKε KO mice after 28 days after angiotensin II (Ang II) (1500 ng/g/day) infusion. **e** Protein levels of p-p38 in remote zone and infarct zone of human failing heart tissues.

Supplementary Fig 4.

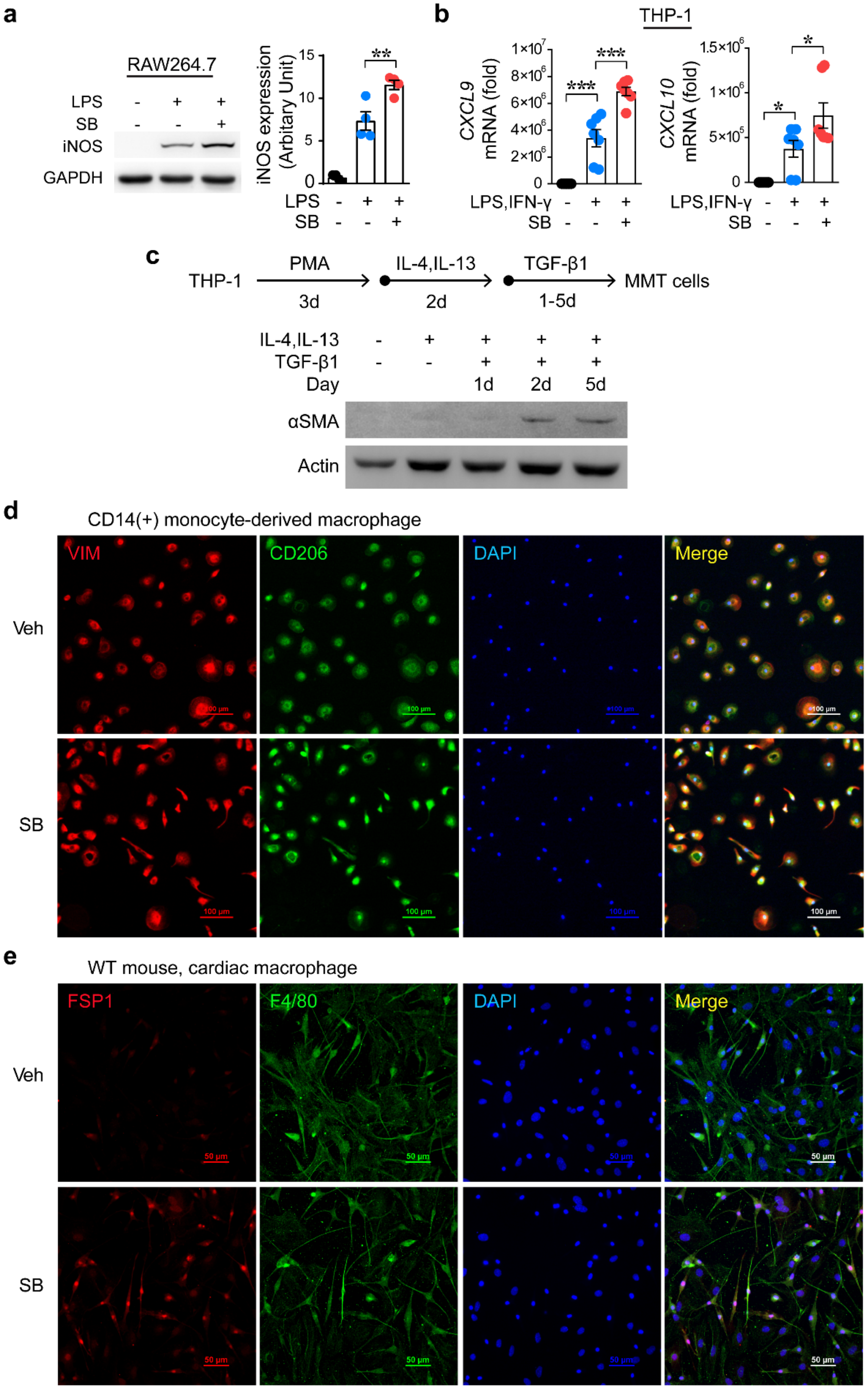

Supplementary Fig 4. Involvement of p38 activity in macrophage-myofibroblast transition (MMT). **a** RAW264.7 cells were stimulated with 100 ng/mL LPS with or without p38 inhibitor 10 μM SB203580 (SB). Induction of iNOS was assessed by Western blot. Protein expression levels of iNOS were quantified. **b** THP-1 cells were stimulated with 100 ng/mL LPS and 30 ng/mL IFN-γ with or without 10 μM SB, Inflammatory markers *CXCL9* and *CXCL10* were assessed by real-time PCR. **c** Induction of αSMA in THP-1 macrophages by MMT induction. **d** CD14(+) mononuclear cells were isolated from peripheral blood to induce differentiation into macrophages. Then cells were treated with 5 ng/mL TGF-β1 with or without 10 μM SB. Immunofluorescence staining showed vimentin (VIM)-expressing CD206(+) anti-inflammatory macrophages. Scale bars: 100μm. **e** Macrophages were isolated from heart tissues at 3 days after myocardial infarction. Then cells were treated with 5 ng/mL TGF-β1 with or without 10 μM SB. Immunofluorescence staining showed FSP1-expressing F4/80(+) macrophages. Scale bars: 50μm. Data are represented as mean ± SEM. $*P < 0.05$; $**P < 0.01$; $***P < 0.001$ (by 1-way ANOVA with Tukey's multiple comparisons test).

Supplementary Fig 5.

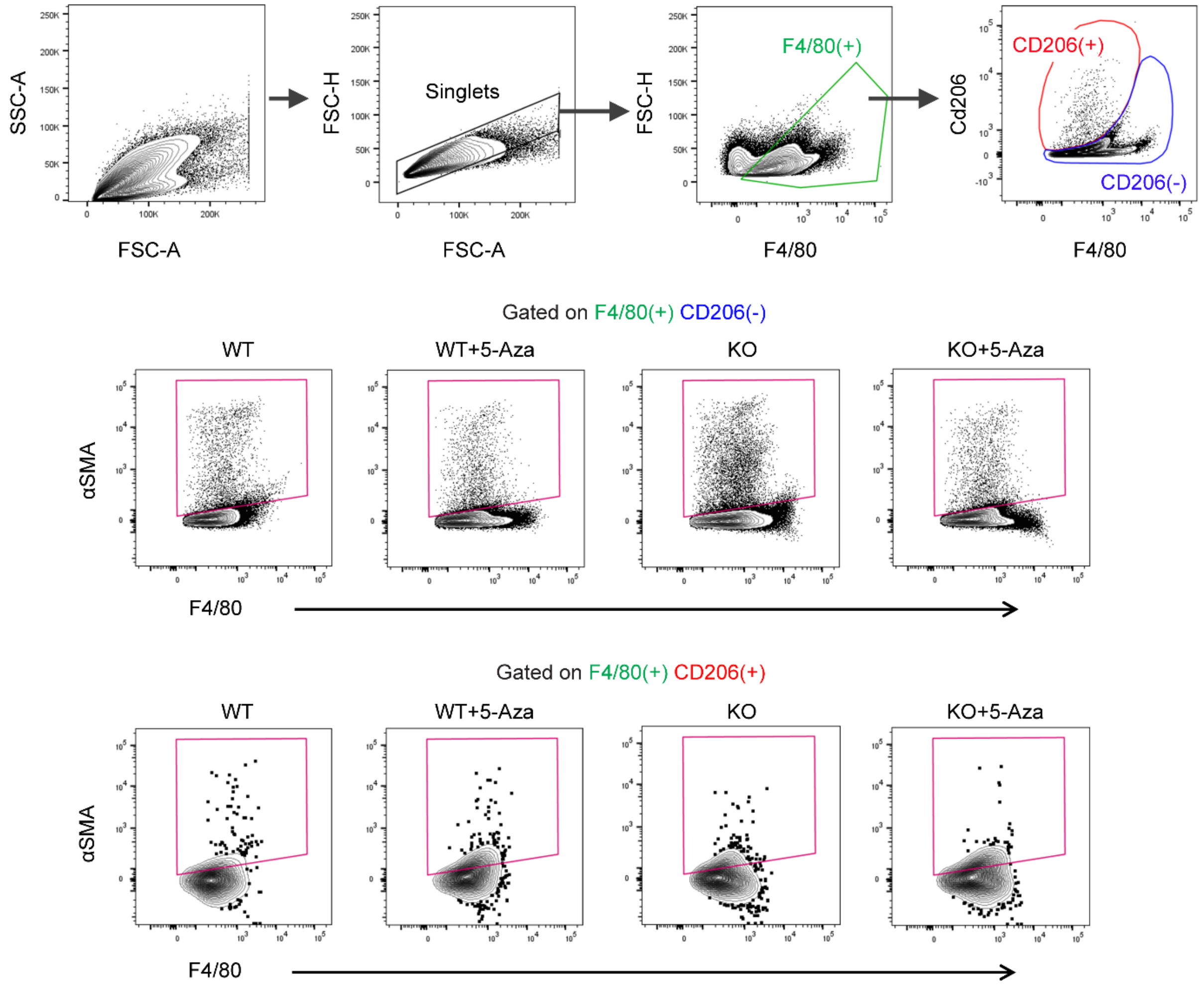

Supplementary Fig 5. Flow cytometry for analyzing cells isolated from infarcted hearts. Myocardial infarction was induced and saline or 5 μg/g 5-azacytidine (5-Aza) were peritoneally injected. Representative gating strategy of macrophage populations in the heart was illustrated.

Supplementary Fig 6.

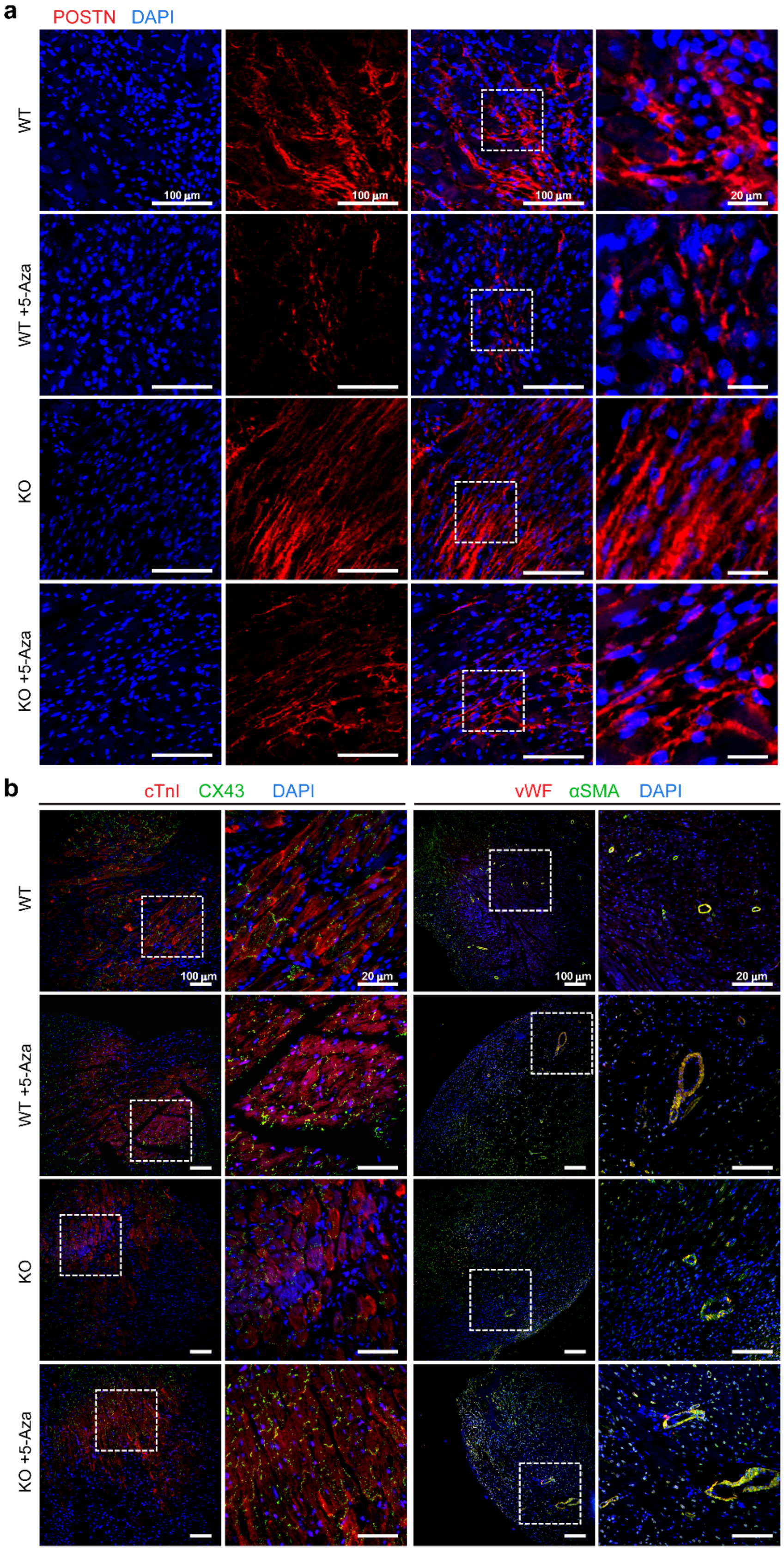

Supplementary Fig 6. Distribution of morphology of fibroblasts, cardiomyocytes, and vessels in the heart tissues at 7 days after myocardial infarction. a The distribution of POSTN(+) fibroblasts was represented in four groups. **b** Aligned connexin 43 (CX43) between cardiac troponin I (cTnI)(+) cardiomyocytes was displayed, and the distribution of blood vessels was represented by Von Willebrand factor (vWF)(+) endothelial cells and αSMA(+) vascular smooth muscle cells.

Supplementary Table 1. The results of echocardiographic analyses at 14 days after MI.

	WT	KO	*p* value
EF (%)	34.02 ± 5.53	30.33 ± 5.25	0.01 **
FS (%)	13.66 ± 2.51	11.99 ± 2.26	0.01 **
IVSd (mm)	0.04 ± 0.01	0.04 ± 0.01	0.08
IVSs (mm)	0.05 ± 0.01	0.04 ± 0.01	0.13
LVIDd (mm)	0.48 ± 0.07	0.48 ± 0.07	0.85
LVPWd (mm)	0.05 ± 0.02	0.05 ± 0.01	0.06
LVPWs (mm)	0.06 ± 0.02	0.05 ± 0.01	0.01 *
EDV (mL)	0.28 ± 0.12	0.29 ± 0.12	0.82
ESV (mL)	0.19 ± 0.09	0.21 ± 0.10	0.46
SV (mL)	0.09 ± 0.04	0.08 ± 0.03	0.25

Supplementary Table 2. Differentially expressed genes for each cell type cluster between IKKε knockout (KO) and wild type mice (WT). (attached excel file)

Supplementary Table 3. The ratio of phosphorylation in bone marrow-derived macrophages according to LPS and IFN-γ stimulation. (attached excel file)

Supplementary Table 4. List of antibodies.

Antibody	Vendor	Catalog No.	Application
PE anti-mouse CD19	BioLegend	152407	Isolation of cardiac cells for flow cytometry
PE anti-mouse Ly6G	BioLegend	127607	
PE anti-mouse NK1.1	BioLegend	108707	
PE anti-mouse TER119	BioLegend	116207	
PE anti-mouse THY1.2	BioLegend	140307	
Alexa fluor 488 anti-alpha smooth muscle actin	abcam	ab184675	Flow cytometry
APC anti-mouse CD206	Invitrogen	17-2061-82	
APC/CY7 anti-mouse F4/80	BioLegend	123117	
FITC anti-mouse CD45	eBioscience	11-0451-82	
Pacific blue ant-mouse Ly6C	BioLegend	128013	
PE anti-mouse CD11b	eBioscience	12-0112-82	
Actin, α-Smooth Muscle	Sigma-Aldrich	A2547	Western blots, immunofluorescence staining
Fibroblast activation protein, alpha	abcam	ab53066	
Phopho-p38 MAPK (Thr180/Tyr182)	CST	9211	
Vimentin	Santa Cruz Biotech	sc-6260	
Actin	Sigma-Aldrich	A4700	Western blots
GAPDH	Santa Cruz Biotech	sc-32233	
IκBα	CST	9242	
IκBβ	LSBio	LS-B397	
IKK gamma/NEMO	Cell Signaling Technology	3416	
IKKG	abcam	ab137363	
IKKα	Santa Cruz Biotech	sc-7218	
IKKβ	CST	2684	
IKKε	CST	3416	
NOS	CST	2977	
p-p65(s468)	CST	3039	
p-p65(s536)	CST	3031	
p105/p50	abcam	ab32360	
p38 MAPK	CST	9212	
p65	Santa Cruz Biotech	sc-7151	

CD68	abcam	ab213363	Immunofluorescence staining
COL1A1	Santa Cruz Biotech	sc-293182	
Connexin 43	Santa Cruz Biotech	sc-9059	
F4/80	abcam	ab6640	
MAC-2 (Galectin-3)	CEDARLANE	CL8942AP	
Periostin	abcam	ab14041	
Periostin/OSF-2	R&D Systems	MAB3548	
Phospho-ATF2 (Thr71)	CST	24329	
S100A4 (FSP1)	abcam	ab197896	
Troponin-I	Santa Cruz Biotech	sc-33728	
vWF	Sigma-Aldrich	F3520	

Supplementary Table 5. Primers for real-time PCR.

Gene	Primer
Human *ACTB*	Forward : ATGCTATCACCTCCCCTGTGTG<br>Reverse : TTGTTACAGGAAGTCCCTTGCC
Mouse *ACTA2*	Forward : TCAGGGAGTAATGGTTGGAATG<br>Reverse : GGTGATGATGCCGTGTTCTA
Mouse *COL1A1*	Forward : GAAACCCGAGGTATGCTTGA<br>Reverse : GGGTCCCTCGACTCCTACAT
Mouse *COL3A1*	Forward : GCACAGCAGTCCAACGTAGA<br>Reverse : TCTCCAAATGGGATCTCTGG
Mouse *COL5A1*	Forward : TTCCAGGCCAAACGGTACAT<br>Reverse : TGAGACACTGTTACAACGATTCCT
Mouse *GAPDH*	Forward : GGGTGTGAACCACGAGAAATA<br>Reverse : GTCATGAGCCCTTCCACAAT
Mouse *iNOS*	Forward : GGAATCTTGGAGCGAGTTGT<br>Reverse : CCTCTTGTCTTTGACCCAGTAG
Mouse *POSTN*	Forward : TGTGTATCGGACGGCTATCT<br>Reverse : CTCTGCTGGTTGGATGATTTCT
Mouse *S100A4*	Forward : TGAACAAGACAGAGCTCAAGG<br>Reverse : AGTTGCTCATCACCTTCTGG

Supplementary materials and methods

Mouse peritonitis model

Peritonitis was induced in male C57BL/6J and IKKε KO mice (8 weeks of age) with a peritoneal injection of 4% thioglycollate (BD Difco, 211716) in 1 mL volume. On the third day, the mice were intraperitoneally injected with 100 μg/0.3 mL LPS (Sigma-Aldrich, L4391) and sacrificed at 4 hours after LPS injection. The peritoneal macrophages were collected from the peritoneal cavity by washing with PBS, and adherent macrophages were assessed by further analyses.

Mouse non-ischemic heart failure model using AngiotensinII infusion

Male C57BL/6J and IKKε KO mice (14 weeks of age) were anesthetized with an intramuscular injection of 50 mg/kg ketamine and 5 mg/kg xylazine. The osmotic mini pump (Alzet 1004) was implanted for 1500 ng/g/day AngiotensinII (Sigma-Aldrich, A9525) infusion under the skin for 4 weeks. Mice in the control group were infused with saline using an Alzet 1004 pump.
